# Gender Inequity Norms Are Associated with Increased Male-Perpetrated Rape and Sexual Risks for HIV Infection in Botswana and Swaziland

**DOI:** 10.1371/journal.pone.0028739

**Published:** 2012-01-11

**Authors:** Kate Shannon, Karen Leiter, Nthabiseng Phaladze, Zakhe Hlanze, Alexander C. Tsai, Michele Heisler, Vincent Iacopino, Sheri D. Weiser

**Affiliations:** 1 Division of AIDS, Department of Medicine, BC Centre for Excellence in HIV/AIDS, University of British Columbia, Vancouver, Canada; 2 Physicians for Human Rights, Cambridge, Massachusetts, United States of America; 3 Department of Nursing, University of Botswana, Gaborone, Botswana; 4 Women and Law in Southern Africa Research Trust, Mbabane, Swaziland; 5 Robert Wood Johnson Health and Society Scholars Program, Harvard University, Cambridge, Massachusetts, United States of America; 6 Department of Internal Medicine, University of Michigan School of Medicine, Ann Arbor, Michigan, United States of America; 7 University of Minnesota Medical School, Minneapolis, Minnesota, United States of America; 8 Positive Health Program, University of California San Francisco, San Francisco, California, United States of America; Tulane University, United States of America

## Abstract

**Background:**

There is limited empirical research on the underlying gender inequity norms shaping gender-based violence, power, and HIV risks in sub-Saharan Africa, or how risk pathways may differ for men and women. This study is among the first to directly evaluate the adherence to gender inequity norms and epidemiological relationships with violence and sexual risks for HIV infection.

**Methods:**

Data were derived from population-based cross-sectional samples recruited through two-stage probability sampling from the 5 highest HIV prevalence districts in Botswana and all districts in Swaziland (2004–5). Based on evidence of established risk factors for HIV infection, we aimed 1) to estimate the mean adherence to gender inequity norms for both men and women; and 2) to model the independent effects of higher adherence to gender inequity norms on a) male sexual dominance (male-controlled sexual decision making and rape (forced sex)); b) sexual risk practices (multiple/concurrent sex partners, transactional sex, unprotected sex with non-primary partner, intergenerational sex).

**Findings:**

A total of 2049 individuals were included, n = 1255 from Botswana and n = 796 from Swaziland. In separate multivariate logistic regression analyses, higher gender inequity norms scores remained independently associated with increased male-controlled sexual decision making power (AORmen = 1.90, 95%CI:1.09–2.35; AORwomen = 2.05, 95%CI:1.32–2.49), perpetration of rape (AORmen = 2.19 95%CI:1.22–3.51), unprotected sex with a non-primary partner (AORmen = 1.90, 95%CI:1.14–2.31), intergenerational sex (AORwomen = 1.36, 95%CI:1.08–1.79), and multiple/concurrent sex partners (AORmen = 1.42, 95%CI:1.10–1.93).

**Interpretation:**

These findings support the critical evidence-based need for gender-transformative HIV prevention efforts including legislation of women's rights in two of the most HIV affected countries in the world.

## Introduction

**“Countries should ensure a massive political and social mobilization to address gender inequities, sexual norms and their roles in increasing HIV risk and vulnerability”**

**– UN Secretary General, Ban Ki- Moon,**

**UN High Level Meeting on HIV/AIDS, April 2008**

Over 33 million people are estimated to be living with HIV worldwide, of whom 70% are in sub-Saharan Africa and 58% are young African women [1]. Among HIV positive adolescents and young adults, age 15–25 years, in sub-Saharan Africa, 70% are female. While 2008 UN global HIV estimates suggest stabilization in the sex-ratio of HIV prevalence in some settings, in many of the highest HIV prevalence countries in the world, such as Botswana and Swaziland, women continue to experience an inequitable burden of new HIV infections [1]. Public health and human rights experts have increasing postulated that systematic gender inequities and women's subordinate position are linked to the alarming HIV epidemics in Southern Africa [2], [3], [4], [5], [6], and a growing body of epidemiological evidence has now shown direct links between sexual coercion, violence, and HIV risk among women and men both in developing and developed country settings [7], [8], [9], [10]. As such, disentangling the underlying sexual and gender norms contributing to violence and risky sex has been identified as critical to designing effective and ‘gender transformative’ HIV prevention programs and policies [11], [12]. Gender transformative HIV prevention programs for both men and women have increasingly been advocated in sub-Saharan Africa as means of addressing sexual and gender inequities in risk of violence and HIV infection [11], [12].

In an intervention trial in Rwanda and cross-sectional samples in South Africa, HIV-positive women were 50% more likely to report intimate partner violence (IPV) than their HIV-negative counterparts [13], [14], even after adjustment for age and sexual risk patterns in the South African sample [7]. A study of women attending an antenatal care clinic in Soweto, South Africa found transactional sex and low sexual decision making power to separately contribute to a 50% increased likelihood of HIV seropositivity (40% HIV prevalence compared to 29% HIV prevalence), regardless of concurrence of sexual or physical violence [15], [16]. Additionally, although research on perpetration of violence and coerced sex among men is relatively scant, recent studies in South Africa, India, and North America have shown that perpetration of partner violence or rape is associated with increased odds of having an sexually transmitted infection (STI) and engagement in higher HIV risk behaviours among men, including multiple concurrent sex partners, transactional sex, sexual assault of non-partners, and use alcohol/or drugs [17], [18], [19]. The separate and independent pathways between violence, power, and HIV infection among women and men suggest different underlying mechanisms may drive these risks. While there is a lack of empirical data on the effects of sexual and gender norms, qualitative research document the perceived “successful” performance of masculinity among high risk men to be predicated on the ability to control and dominate women, in the context of entrenched gender inequity norms [20], [21], [22]. Theoretical work conceptualizes ‘gender inequity norms’ as adherence to socially and culturally embedded norms on gender and sexual roles among men and women, including expectations on masculinity and femininity [12], [20], [21], [22], [23]. Understanding how adherence to gender inequity norms may shape violence and HIV risks can help to identify pathways for targeting, gender-focused interventions [23], [24].

Using a large population-based sample in Botswana and Swaziland, this study examines adherence to gender inequity norms among men and women and the separate epidemiological relationship with established HIV risk factors: a) male sexual dominance (male-controlled sexual decision making, rape (forced sex); and b) interpersonal sexual risks practices (unprotected sex with non-primary partners, multiple/concurrent sex partners, transactional sex, intergenerational sex). Though some important advancements have been made in civil legislation, customary laws in Botswana and Swaziland continue to promote women's subordination to men, and critical gaps remain surrounding domestic violence legislation.

## Methods

### Population Setting

Data were derived from a population-based cross-sectional study conducted in Botswana and Swaziland between November 2004 and May 2005. Eligibility criteria were adults of reproductive age (18–49 years) who were residents of the country, and were fluent in either English or siSwati or Setswana (the most common local languages). The methods have been previously described in detail elsewhere [25]. Briefly, based on the assistance of the local in-country Central Statistics Offices, a stratified two-stage probability sampling design was used to randomly select individuals from households in all four districts in Swaziland and in the five districts with the highest HIV prevalence in Botswana (Gaborone, Kweneng East, Francistown, Serowe/Palapye, Tutume). Within each household, 1 adult member for whom the house was his or her primary residence and who met the study's inclusion criteria was randomly selected for inclusion in the study. Up to 2 repeat attempts were made to interview that person if the initial visit was unsuccessful. No replacements were made if participants could not be reached after the repeat attempts. We did not interview more than 1 member of the household. Study procedures were approved by a number of ethical review boards, including Botswana Ministry of Health Research and Development Committee, Ethics Committee of Swaziland Ministry of Health, University of California San Francisco, and Physicians for Human Rights.

### Data Collection

A structured interview-administered questionnaire asked questions related to demographics, sexual risk patterns, violence, HIV stigma, and measures of gender inequity norms. All surveys and consent forms were translated into the local language (either Setswana or siSwati) and back-translated into English by the study team (country nationals). All interviews were conducted in private settings, and anonymity was assured. The field research team consisted of country nationals with prior research experience (most in the area of HIV/AIDS), who were trained by a team of Physicians for Human Rights research staff along with local researchers. The supervisory team had extensive expertise in applied research, human rights, gender issues, mental health, and HIV/AIDS. The training included detailed instruction in the study protocols and research ethics and field practice in interviewing. The survey team received specific training on how to enumerate households (e.g., not counting nonresidential buildings, counting each separate household on the same property separately) and how to ask sensitive questions in an appropriate, nonjudgmental manner. Participants who experienced any emotional distress during the course of the interview were offered the opportunity to speak to one of the study health care providers, in addition to referral to local health care center for counseling. All participants were offered literature regarding HIV/AIDS testing, prevention, and treatment, and information concerning domestic violence. They were also offered information on how to report domestic violence and rape to local enforcement in accordance with national laws.

### Gender Inequity Norms

Our primary explanatory variable was an index of ‘gender inequity norms’, consisting of six measures of gender inequity norms (Table 1) developed based on our qualitative research in this setting and previous theory and research in the peer-review literature [12], [20], [21], [22]. As described above, qualitative research and theoretical work conceptualizes ‘gender inequity norms’ as adherence to socially and culturally embedded norms on gender and sexual roles among men and women, including expectations on gender roles, access to resources (education, inheritance) and adherence to traditional concepts of masculinity and femininity. We calculated the Cronbach's alpha to demonstrate the internal reliability of our scale. A scale is generally considered reliable if the Cronbach's alpha coefficient is equal to or greater than 0.70, and for exploratory studies, a coefficient of ≥0.60 is considered acceptable. The Cronbach's alpha value was 0.75. Eight additional measures loading less than 0.35 were eliminated (such as “A woman must prove her fertility before she can marry”) leaving these final six measures to comprise the gender inequity norms index. In sensitivity analyses, we found similar trends in associations with our HIV risk outcomes using a more conservative index of gender inequity norms that excluded the two measures related to violence (*results not shown*). Individual scores were entered into regression models, with increasing values indicating greater adherence to gender inequity norms. Mean gender inequity norm scores were calculated separately for men and women.

**Table 1:** Measures for gender inequity norms index among men and women.

“It is ok for men to have more than one (sexual) partner”
“It is a woman's duty to have sex with her spouse/partner even if she does not want to”
“It is more important for a woman to respect her spouse/partner than it is for a man to respect his spouse/partner”
“A man may beat this spouse/partner if she disobeys him”
“A man may beat this spouse/partner if he believes she is having sex with another man”
“It is more important for a boy to get an education than a girl”

### Sexual Power and HIV Risk Measures

Based on research of established risk factors for HIV infection in sub-Saharan Africa and theoretical concepts of sexual power and HIV [7], [8], [9], [10], [26], we examined two outcomes to capture male sexual dominance and four measures of sexual risk practices: 1) **Male-controlled sexual decision making** –defined based on a power differential in response to the following two questions: “Who generally decides when you have sex?” and “In your sexual encounters, who usually decides whether you use a condom.?”. Male-controlled sexual decision-making has been conceptualized as sexual relationship power to better capture the sexual division of power and negotiation of sexual risk practices among men and women in the context of HIV [5], [26], [27]. For our analyses, among women, male-controlled sexual decision making was defined as a response of “mostly your partner” or “partner only” to one or both questions, and among men, as a response of “mostly you” or “only you”.; 2) **Rape (forced sex)** - a) ***perpetration of rape*** -defined among men as a ‘yes’ response to “have you had sex with others when they did not want to?”, consistent with recent work [28]. b) ***rape*** defined among women as a ‘yes’ response to: “Were you forced to have sex against your will?”. 3) **Intergenerational sex** - defined among men as having a partner at least 10 years younger; and among women, as having a partner at least 10 years older. 4) **Transactional sex** - defined among men as providing money or resources to a partner in exchange for sex; and among women, defined as receiving money or resources (e.g. food, child support) from a partner in exchange for sex; 5) **Unprotected sex with non-primary partners** - defined as inconsistent condom use with a non-primary partner; 6) **Multiple/concurrent partners** –defined as having more than one sex partner. All measures used a recall period of the previous 12 months.

### Covariates

Based on previous research [7], [8], [9], [10], socio-demographic variables considered *apriori* as potential confounders of the relationship between gender inequity norms and our outcomes of interest included: age (continuous, per year), relationship status (defined as single, married, or cohabitating), education (≥high school vs. <high school education), annual household income (dichotomized at the ordinal variable closest to the sample median in each country), rural residence (vs. urban), and risky alcohol use (defined as heavy drinking, problem drinking vs. moderate/no drinking using the National Institute of Alcohol Abuse and Alcoholism definitions).

### Statistical Analyses

One-way ANOVA tests were used to examine differences in mean gender inequity scores by socio-demographic characteristics and each of our outcomes. Bivariate analyses were conducted to obtain crude odds ratios for the relationship between explanatory variables and each of our outcomes of interest and to test for potential collinearity. Given hypothesized sex differences in risky pathways for gender inequity norms, all bivariate and multivariate analyses were stratified by sex (male/female). Separate multivariate logistic regression models were constructed to obtain adjusted affects of the relationship between mean scores for gender inequity norms and each of the outcome measures, controlling for potential confounders and variables significant in bivariate analyses. Given the small sample size for some measures, we used a p-value cut-off of <0.10 for entry into our model. All reported p-values are 2-sided and odds ratios (ORs) are reported with 95% confidence intervals (CIs). All statistical analyses were performed using SAS software version 9.1 (SAS, Cary, North Carolina). Given that each of our outcomes were modeled separately, crude and adjusted odds ratios of the relationship between explanatory variables and outcomes of interest are reported separately. Consistent with previous work, to account for likely heterogeneity of responses between countries, we adjusted all multivariate models for country of recruitment. In addition, we conducted country-specific models to evaluate the trends in associations between our mean gender inequity norms and HIV risk outcomes in each setting. Given that the same trends in associations were observed for all our outcome measures in country-specific models, we report the results for the global model, controlling for differences by country.

## Results

A total of 2049 individuals of reproductive age (15–49 years) were included in the analyses (response rate of 89%), 1255 individuals from Botswana and 796 individuals from Swaziland. Table 2 provides the sociodemographic data and prevalence of sexual risk practices and violence, stratified by men and women in both Botswana and Swaziland. As indicated, the median age of the total sample was 27 years (IQR = 22–35) and 1050 (51%) were women. Fifty-three percent of women were married or cohabitating, and 44% of men. Approximately half had a high school education or higher (47% of women; 52% of men) and one-third (33% of women; 38% of men) were living in rural communities. Among women, 274 (26%) reported male-controlled sexual decision-making (e.g. partner decides when/how often to have sex and/or when to use condoms) and 49 (5%) reported being raped (forced sex) in the previous 12 months (See table 1). Among men, 432 (41%) reported male-controlled sexual decision-making, and 33 (3%) reported perpetration of rape (forced sex) in the previous 12 months. In terms of sexual risk practices in the previous 12 months, 402 (39%) men reported having multiple/concurrent sex partners (mean = 6, median = 3, IQR: 2–3), 151 (15%) reported intergenerational sex with a 10+ years younger woman, 113 (11%) unprotected sex with non-primary partners, and 96 (10%) provided money or resources in exchange for sex. Among women, 198 (18%) reported multiple/concurrent sex partners (mean = 5, median = 2, IQR: 2–2), 177 (17%) intergenerational sex with a male partner 10+ years older, 77 (8%) reported unprotected sex with non-primary partners, and 50 (5%) had received money or resources in exchange for sex. The mean score for gender inequity norms for women was 1.5 (−5 to 1.8) and for men was 1.0 (−4 to 2.6). Table 1 shows the measures included in the gender inequity norms index. Overall, men in Swaziland had higher gender inequity norms scores than those in Botswana (p<0.001), while there were no statistically differences in gender inequity norms among women between the two countries (p = 0.356). Both men and women with less than high school education and lower monthly household income had a higher mean gender inequity norms scores (p<0.010), as did married men (p<0.004) and women living in rural communities (p = 0.020) compared to single/cohabitating men and women in urban centres.

**Table 2:** Socio-demographic characteristics, sexual risk practices and male sexual dominance among men (n = 999) and women (n = 1050) in a population-based probability sample in Botswana and Swaziland.

		Women (n = 1050)	Men (n = 999)
		n(%)	n(%)
**Age, years** (median, IQR)		26.9 yrs (21–34)	28.1 yrs (23–36)
**Relationship Status**	Single	493 (47%)	561 (56%)
	Cohabitating	264 (25%)	223 (22%)
	Married	293 (27%)	215 (22%)
**Education**	High School or Higher	603 (57%)	539 (52%)
	Less than High School	447 (43%)	460 (48%)
**Monthly Household Income**	Greater than 5000 Pula (Botswana) or 5000 emalangeni (Swaziland) = US$800–1000	366 (35%)	304 (30%)
**Alcohol Use**	Problem or heaving drinking	177 (17%)	289 (29%)
**Residence**	Urban (vs. rural residence)	699 (67%)	649 (65%)
**Mean Gender Inequity Norms Score**		1.5 (−5 to 1.8)	1.0 (−4 to 2.6)
**Male sexual dominance** (past year)	Male-controlled sexual decision making	276 (26%)	432 (41%)
	Perpetrated rape (forced sex)	----	33 (3%)
	Raped (forced sex against your will)	49 (5%)	----
**Sexual Risk Practices** (past year)	Multiple/concurrent sex partners	198 (19%)	402 (39%)
	Number of sex partners (median, IQR)*	2 (2–2)	3 (2–3)
	Intergenerational sex (10+ years age difference)	177 (17%)	151 (15%)
	Transactional sex (exchange of sex for money or other basic resources)	50 (5%)	96 (10%)
	Unprotected sex with a non-primary sex partner	77 (8%)	113 (11%)

Tables 3 and 4 show the separate bivariate associations between gender inequity norms scores and each of our violence and sexual risk measures among women and men respectively. All tests of collinearity were non-significant. Higher gender inequity norms scores were significantly associated with lower control over sexual decision-making, transactional sex and intergenerational sex among women in unadjusted analyses (Table 3). Among men (Table 4), higher gender inequity norm scores were significantly associated with male-controlled sexual decision-making, perpetration of rape, unprotected sex with a non-primary partner and multiple sexual partners in unadjusted analyses.

**Table 3:** Crude odds ratios of associations between gender inequity norms scores and rape, power, and sexual risk for HIV infection among women in Botswana and Swaziland (n = 1050).

Violence, Power, and Sexual Risk Outcomes							
Characteristic		Low control over sexual decision-making	Rape(forced sex)	Transactional Sex (Received money, food or other resources in exchange for sex)	Intergenerational Sex (partner 10+ years older)	Unprotected sex with non-primary partners	Multiple/concurrent sex partners
**Age** (continuous +1 yr)		**1.02 (1.00–1.04)**	1.03 (0.98–1.06)	0.99 (0.99–1.00)	1.02 (0.99–1.04)	0.97 (0.95–0.98)	1.04 (0.96–1.09)
**Relationship Status**	Single	Reference	Reference	Reference	Reference	Reference	Reference
	Cohabitating	**2.10 (1.63–2.69)**	**0.75 (0.41–0.92)**	**2.57 (2.44–2.70)**	**1.58 (1.05–2.37)**	1.56 (0.80–3.02)	0.82 (0.71–1.13)
	Married	**2.12 (1.65–2.74)**	0.89 (0.54–1.41)	**0.53 (0.44–0.64)**	1.10 (0.71–1.70)	0.67 (0.35–1.30)	**0.65 (0.41–0.78)**
**Education**	≥High School	**0.31 (0.30–0.33)**	0.96 (0.53–1.73)	**0.62 (0.58–0.67)**	**0.68 (0.53–0.86)**	0.91 (0.71–1.16)	0.78 (0.57–1.06)
	<High School	Reference	Reference	Reference	Reference	Reference	Reference
**Monthly Household Income**		**0.57 (0.55–0.60)**	1.03 (0.56–1.90)	0.64 (0.20–2.06)	0.72 (0.62–8.43)	1.14 (0.38–3.51)	0.89 (0.64–1.23)
**Mean Gender Inequity Norm**		**2.10 (1.59–2.73)**	0.91 (0.77–1.13)	**1.67 (1.09–1.98)**	**1.40 (1.11–2.75)**	1.24 (0.90–1.79)	0.99 (0.85–1.10)
**Alcohol Use** *	None to Moderate	Reference	Reference	Reference	Reference	Reference	Reference
	Problem Drinking	1.18 (0.90–1.54)	**1.97 (1.17–2.76)**	**10.99 (6.47–18.69)**	**2.06 (1.74–2.45)**	**3.98 (1.18–13.47)**	**2.98 (1.87–3.41)**
	Heavy Drinking	1.13 (0.70–1.81)	**3.94 (2.19–7.12)**	**15.59 (4.51–53.82)**	**3.84 (2.87–5.13)**	**7.02 (1.81–27.20)**	**5.11 (3.60–7.30)**
**Country of Recruitment**	Botswana	0.68 (0.52–0.88)	0.47 (0.10–2.18)	**5.81 (2.29–14.67)**	**1.43 (1.01–2.02)**	0.51 (0.39–0.67)	**6.69 (3.05–14.71)**
	Swaziland	Reference	Reference	Reference	Reference	Reference	Reference
**Residence**	Rural	1.37 (0.79–2.38)	1.13 (0.62–2.06)	1.68 (0.53–5.35)	**1.30 (1.14–1.48)**	0.49 (0.31–0.76)	0.84 (0.57–1.29)
	Urban	Reference	Reference	Reference	Reference	Reference	Reference

*Based on National Institutes of Alcohol Use definition of “risky drinking”, problem drinking was defined as 8–14 drinks/week for women and 15–21 drinks/week for men, while heavy drinking was defined as >14 drinks/week for women and >21 drinks/week for men.

**Table 4:** Crude odds ratios of associations between gender inequity norms score and perpetration of rape, power, and sexual risk for HIV infection among men in Botswana and Swaziland (n = 999).

Violence, Power, and Sexual Risk Outcomes							
Characteristic		Male-controlled sexual decision-making	Perpetration of rape(forced sex)	Transactional Sex (Provided money, food or other resources in exchange for sex)	Intergenerational Sex (partner 10+ years younger)	Unprotected sex with non-primary partners	Multiple/concurrent sex partners
**Age** (continuous +1 yr)		**1.13 (1.06–1.19)**	1.04 (0.96–1.10)	1.06 (0.89–1.14)	1.10 (1.06–1.14)	1.02 (1.00–1.05)	1.01 (0.96–1.07)
**Relationship Status**	Single	Reference	Reference	Reference	Reference	Reference	Reference
	Cohabitating	**2.03 (1.56–2.79)**	1.37 (0.92–1.71)	0.93 (0.73–1.19)	0.77 (0.39–1.52)	1.25 (0.64–2.45)	0.92 (0.82–1.34)
	Married	**1.89 (1.24–2.36)**	1.26 (0.85–1.64)	**0.19 (0.06–0.58)**	0.31 (0.08–1.29)	0.79 (0.40–1.56)	**0.75 (0.53–0.91)**
**Education**	≥High School	Reference	Reference	Reference	Reference	Reference	Reference
	<High School	**0.62 (0.48–0.80)**	1.55 (0.75–3.21)	1.12 (0.80–1.57)	0.82 (0.39–1.75)	0.90 (0.57–1.40)	0.93 (0.73–1.21)
**Monthly Household Income**		**0.48 (0.37–0.63)**	1.22 (0.62–2.48)	1.94 (1.59–2.37)	1.07 (0.37–3.09)	**0.78 (0.72–0.84)**	1.00 (0.78–1.30)
**Mean Gender Inequity Norm**		**1.98 (1.17–2.60)**	**2.55 (1.20–3.89)**	1.08 (0.86–1.87)	1.05 (0.93–1.27)	**2.01 (1.29–2.97)**	**1.25 (1.14–1.97)**
**Alcohol Use** *	None to Moderate	Reference	Reference	Reference	Reference	Reference	Reference
	Problem Drinking	1.05 (0.73–1.28)	**1.94 (1.29–2.51)**	**3.22 (1.87–5.54)**	1.04 (0.51–2.13)	**2.09 (1.35–3.22)**	**1.98 (1.66–2.78)**
	Heavy Drinking	1.28 (0.97–1.67)	**3.78 (1.85–7.70)**	**3.87 (3.27–4.59)**	**1.95 (1.67–2.27)**	**2.63 (1.66–4.15)**	**2.86 (2.16–3.79)**
**Country of Recruitment**	Botswana	**0.43 (0.33–0.55)**	**2.40 (1.03–5.58)**	**3.21 (1.87–5.50)**	0.92 (0.65–1.31)	0.48 (0.37–0.62)	1.02 (0.79–1.32)
	Swaziland	Reference	Reference	Reference	Reference	Reference	Reference
**Residence**	Rural	1.19 (0.92–1.53)	0.50 (0.22–1.12)	1.13 (0.57–2.24)	1.11 (0.69–1.32)	1.06 (0.31–3.65)	0.84 (0.65–1.10)
	Urban	Reference	Reference	Reference	Reference	Reference	Reference

*Based on National Institutes of Alcohol Use definition of “risky drinking”, problem drinking was defined as 8–14 drinks/week for women and 15–21 drinks/week for men, while heavy drinking was defined as >14 drinks/week for women and >21 drinks/week for men.

As indicated in Table 5, in sex-stratified models using multivariate logistic regression and adjusting for potential confounders, higher gender inequity norms scores remained independently associated with increased male-controlled sexual decision making power (AORmen = 1.90, 95%CI:1.09–2.35; AORwomen = 2.05, 95%CI:1.32–2.49), indicating that as gender inequity norms increase, men are more likely to control sexual decision making. In adjusted analyses, gender inequity norms scores were positively associated with perpetration of rape (AORmen = 2.19 95%CI:1.22–3.51), unprotected sex with a non-primary partner (AORmen = 1.90, 95%CI:1.14–2.31), intergenerational sex (AORwomen = 1.36, 95%CI:1.08–1.79), and multiple/concurrent sex partners (AORmen = 1.42, 95%CI:1.10–1.93).

**Table 5:** Adjusted odds ratios of the independent relationship between gender inequity norms scores and violence, power, and sexual risk practices in Botswana and Swaziland, in sex-stratified models.

Violence, Power, and Sexual Risk Outcomes								
Gender Inequity Norms	Sex-Specific Models	Male-controlled sexualdecision-making	Rape Perpetration(forced sex)	Raped(forced sex)	Transactional Sex	Intergenerational Sex (10+ years)	Unprotected sex with non-primary partners	Multiple/concurrent sex partners
		**AOR**						
	**Men**	**1.90**	**2.19**	-------	1.12	1.06	**1.90**	**1.42**
		**(1.09–2.35)** **	**(1.22–3.51)** **	-------	(0.83–1.89)	(0.78–1.59)	**(1.14–2.31)** **	**(1.10–1.93)** **
	**Women**	**2.05**	--------	0.83	1.35	**1.36**	1.35	0.79
		**(1.32–2.49)** **	--------	(0.42–1.59)	(0.99–1.64)*	**(1.08–1.79)** **	(0.86–2.21)	(0.51–1.59)

All models adjusted for age, married/cohabitating, country of residence, alcohol consumption, and variables significant at p<0.10 in univariate analyses;

**Variables retained at significance p<0.05.

*Variables marginally significant at p<0.10.

## Discussion

Our findings demonstrate that greater adherence to gender inequity norms both in Botswana and Swaziland is associated with increased male sexual dominance, perpetration of rape and sexual risk practices. Given that male sexual dominance and risky sex have been previously established as risk factors for HIV infection among men and women [7], [8], [9], [10], these findings support growing calls for gender-transformative HIV prevention efforts [11], [29] including legislation to ensure women's rights in two of the most HIV affected countries in the world.

More specifically, our study demonstrates that higher adherence to gender inequity norms are associated with elevated women's risk of HIV acquisition by reducing women's control over their sexual and reproductive health (including use of barrier contraceptives, decisions on when/how often to have sex), and simultaneously increasing economic dependence on men through intergenerational sex with older men and transactional sex. Previous research in sub-Saharan Africa has consistently demonstrated that financial or material dependence on men for basic resources (e.g. food, child care) in exchange for sex introduces power differentials in negotiations over sex and condom use that places women at increased risk for HIV infection. Importantly, while there is scant empirical evidence on the effects of gender inequity norms, our study results contrast with a recent report among youth suggesting strong gender inequity norms differentially impact condom use for men and women. Specifically, among a small sample of youth (18–24 years) in secondary school in northern KwaZulu/Natal, South Africa, adhering to gender inequity norms was correlated with increased condom use among men and conversely, reduced condom use among women [23]. Comparatively, in our study, strong gender inequity norms among men had the reverse association, and had no statistically significant effect on condom use among women. Instead the consistent pathway for both men and women between gender inequity norms and male-controlled sexual decision-making extends earlier work of the underlying mechanisms shaping male sexual dominance and women's HIV risk.

Of particular importance, our findings demonstrate that in two countries where marital rape is not criminalized, and laws continue to promote women's subordinate position in society, men who adhere to gender inequity norms are at two-fold increased odds of male sexual dominance and rape, and are also more likely to engage in HIV risk practices such as unprotected sex and having multiple sexual partners. While research on perpetration of rape among men in sub-Saharan Africa remains extremely limited, two important studies of young men South Africa recently documented a relatively high prevalence of both intimate and non-intimate partner violence [17], [28]. The authors hypothesized that perpetration of rape is related to men's desire to seek power and control over women and confirm male's subordinate position over women. Our study therefore provides critical evidence to confirm this hypothesis [28].

In light of a recent study by UNICEF and US Centers for Disease Control demonstrating one-third of adolescent girls in Swaziland had experienced sexual violence (including rape) before 18 years of age, these results contribute to the evidence-based need for greater involvement of boys and men in gender-transformative HIV prevention efforts, as articulated in Cairo at the International Conference on Population and Development (ICPD) in 1994 [30]. The Stepping Stones Trial in the Eastern Cape of South Africa demonstrates significant potential for ‘gender-transformative HIV prevention’. Adolescent boys and young men who were more resistant to peer pressure to have sex and held more equitable gender attitudes were at reduced likelihood for sexually transmitted infections (STIs), perpetration of violence, and providing financial resources in exchange for sex [31]. The results, however, found no significant impact on risk patterns for women or HIV incidence in either group, suggesting continued need to disentangle the links between underlying gender inequity norms and specific HIV risk practices.

Furthermore, our findings extend the body of evidence demonstrating both a public health and human rights imperative of effectively ending gender discrimination in civil, political and economic laws, including reforms to marriage, inheritance, property and employment laws in Swaziland and Botswana [2], [3]. In particular, civil and customary laws need to be commensurate with the provisions of international human rights covenants and conventions that both Botswana and Swaziland have ratified. For example, in light of current legislative barriers to control over financial resources among women in Botswana and Swaziland, this study extends early research supporting the critical need to legislate women's equal access to economic resources, including education opportunities, credit, land ownership and inherited property. Furthermore, laws need to be harmonized with the Women's Convention of Cairo which protects the sexual and reproductive rights of women, including control over the number and spacing of children and access to family planning [32]. Unfortunately, while the appointment in 2003 of a UN Secretary General's Task for on Women, Girls and HIV/AIDS in Southern Africa demonstrated the increasing recognition of the links between gender inequity and the HIV pandemic, human rights concerns continue to be sidelined in national actions plans and HIV policies [33].

There are several limitations that need to be considered when interpreting our results. This study is cross-sectional in nature and cannot assess temporality and thus casual relationships cannot be drawn. In addition, due to self-report bias, it is likely that measures of male sexual dominance, rape, and sexual risk-taking were likely underestimated. Our measure of power was based on two questions, and therefore likely does not capture the full range of decision-making and male dominance elicited in the validated Sexual Relationship Power Scale (SRPS). However our two measures were separately validated questions incorporated into the SRPS. Additionally, our measure of perpetration of rape did not distinguish between primary and non-primary partners which have been shown to have different HIV risk pathways [17], [28], and does not explicitly ask about individual rape experiences versus gang rape. However our study offers evidence to suggest the underlying mechanism by which gender inequity norms shape perpetration of rape. Furthermore, the recall period of 12-months likely underestimates violence experienced over a lifetime. Our measure of transactional sex did not distinguish between informal exchange of sex for resources and more formal commercial sex work. Finally, further measures and analysis are needed to try to capture the macro-level effects of legislation and their downstream effects on gender inequity norms, violence and HIV risk.

In summary, our findings suggest that a failure to effectively promote gender equality may continue to have dramatic effects on shaping the HIV epidemic through gendered sexual risk patterns and perpetration of male sexual dominance and violence against women. The global call by UN Secretary General, Ba Ki-Moon for countries to systematically end gender inequalities at the UN High Level Meeting in 2008, coupled with the recent establishment of a high-level UN agency focused on women's rights, suggest a critical momentum for gender-transformative HIV prevention that prioritizes women's rights on the global HIV agenda.
